# Aggressive Pituitary Macroadenoma Treated With Capecitabine and Temozolomide Chemotherapy Combination in a Patient With Nelson’s Syndrome: A Case Report

**DOI:** 10.3389/fendo.2021.731631

**Published:** 2021-11-11

**Authors:** Oriol Mirallas, Francesca Filippi-Arriaga, Irene Hernandez Hernandez, Anton Aubanell, Anas Chaachou, Alejandro Garcia-Alvarez, Jorge Hernando, Elena Martínez-Saez, Betina Biagetti, Jaume Capdevila

**Affiliations:** ^1^ Medical Oncology Department, Gastrointestinal and Endocrine Tumor Unit, Vall d’Hebron University Hospital and Vall d’Hebron Institute of Oncology (VHIO), Barcelona, Spain; ^2^ Clinical Pharmacology Department, Vall d’Hebron University Hospital, Barcelona, Spain; ^3^ Endocrinology & Nutrition Service, Vall d’Hebron University Hospital, Barcelona, Spain; ^4^ Radiology Department, Vall d’Hebron University Hospital, Barcelona, Spain; ^5^ Pathology Department, Vall d’Hebron University Hospital, Barcelona, Spain

**Keywords:** capecitabine, temozolomide, aggressive pituitary tumors, Nelson’s syndrome, case report

## Abstract

Nelson’s syndrome is considered a severe side effect that can occur after a total bilateral adrenalectomy in patients with Cushing’s disease. It usually presents with clinical manifestations of an enlarging pituitary tumor including visual and cranial nerve alterations, and if not treated, can cause death through local brain compression or invasion. The first therapeutic option is surgery but in extreme cases of inaccessible or resistant aggressive pituitary tumors; the off-label use of chemotherapy with capecitabine and temozolomide can be considered. However, the use of this treatment is controversial due to adverse events, lack of complete response, and inability to predict results. We present the case of a 48-year-old man diagnosed with Nelson’s syndrome with prolonged partial response and significant clinical benefit to treatment with capecitabine and temozolomide.

## Introduction

Nelson’s syndrome (NS) is characterized by an elevation of adrenocorticotropic hormone (ACTH), hyperpigmentation, and an expanding pituitary mass. NS is considered a severe side effect of total bilateral adrenalectomy (TBA) that occurs as a consequence of missing glucocorticoid feedback to control adenoma cells in patients with Cushing’s disease (CD), which results in an invasive macroadenoma of the pituitary gland (1). CD represents around 70% of the forms of chronic endogenous hypercortisolism and is caused by excessive secretion of cortisol from the adrenal glands secondary to stimulation of an ACTH-producing pituitary tumor (2). CD has a prevalence of 40 cases per million with an incidence from 1.2 to 2.4 per million per year (2, 3). Usually, CD presents in the fourth to sixth decade of life and is more common in women than in men with a ratio of 3:1 (2, 3). Almost 10% of patients with CD eventually undergo TBA and the incidence of NS in patients with TBA shows a high variation, ranging from 0% to 47% with a median of 21% at a median follow-up of 61 months (1). The first-line treatment for CD is transsphenoidal surgery since it is the only curative treatment. When surgery fails, other options include repeating surgery (if feasible), radiotherapy, bilateral adrenalectomy, and/or medical treatment (4, 5). Treatment with TBA is performed in patients in whom all other treatment options have failed; the advantage of this procedure is usually the immediate control of hypercortisolism (1). The criteria for the diagnosis of NS include a plasma ACTH level above 200 ng/L, imaging evidence of pituitary mass enlargement, and hyperpigmentation (4). If a patient develops NS after TBA, the primary treatment is surgery, but in patients with inaccessible or resistant aggressive pituitary tumors, the off-label use of chemotherapy or the experimental use of peptide receptor radionuclide therapy (PRRT) may be considered (6, 7). Treatment with temozolomide (TMZ) alone has demonstrated favorable responses in some case reports against a variety of aggressive pituitary tumors (8). However, the use of this treatment can be controversial due to adverse events and lack of complete response to it. In some cases, the addition of capecitabine (CAP) has been proposed; however, the effect of the combination of these drugs in NS has been less defined and the long-term repercussions are unknown (9). Untreated NS adenomas often become markedly aggressive and may cause death, usually through local brain compression or invasion (5). Herein, we present the case of a Nelson’s syndrome patient treated with CAP-TMZ, who achieved a prolonged partial response with great tolerance.

## Case Presentation

**Figure 1:**
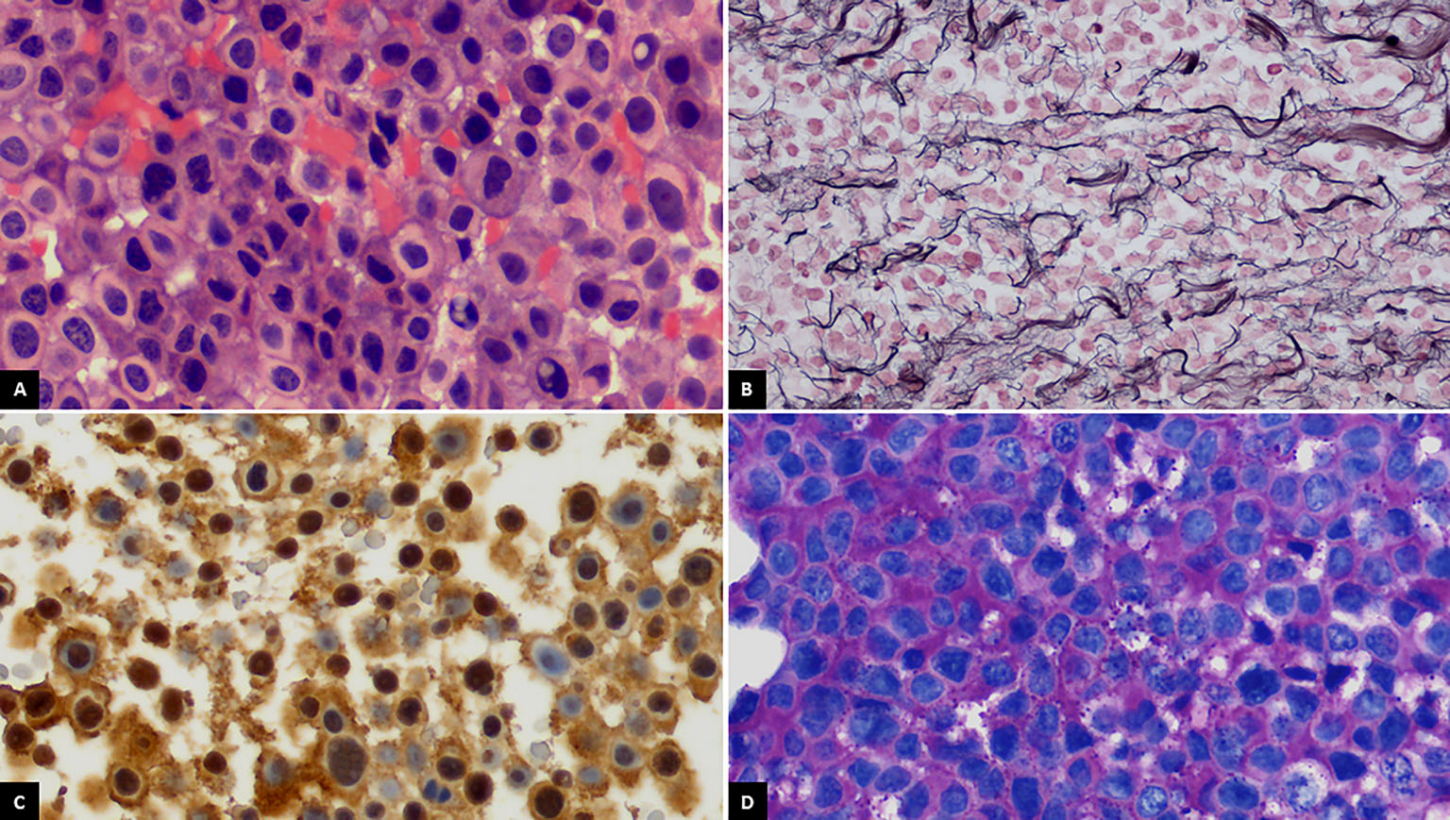
**(A)** Crooke cells exhibiting wide glassy pale eosinophilic cytoplasm with intranuclear or perinuclear vacuoles (H&E, ×400). **(B)** Breakdown of reticulin network in the lesion (Gordon’s reticulin, ×400). **(C)** ACTH cytoplasmic immunoreactivity at the periphery of the cell (ACTH, ×400). **(D)** Periodic acid-Schiff (PAS) positivity staining stronger at the periphery of the cell (×400).

A 48-year-old man was referred to our hospital for the study of cushingoid phenotype. Medical history included allergy to penicillin and dyslipidemia. He presented an ACTH of 152 pg/ml (range, 4.7–48.8 pg/ml) cortisol of 47 μg/dl (range, 5.27–22.45 μg/dl), dynamic endocrine laboratory tests were suggestive of CD, and the magnetic resonance imaging (MRI) revealed a macroadenoma. Therefore, initial treatment with a transsphenoidal resection was performed. The morphological changes were consistent with a pituitary adenoma, with a loss of reticulin network (**Figure 1B**). The cells displayed a pathological appearance with a glassy pale eosinophilic cytoplasm, with intranuclear or perinuclear vacuoles, consistent with Crooke’s hyaline change (**Figure 1A**). ACTH cytoplasmic immunoreactivity was found at the periphery of the cell (**Figure 1C**); PAS positivity was found, stronger at the periphery (**Figure 1D**). The proliferation index (Ki-67) reached 2% to 4% of cells. Pituitary surgery was unsuccessful, and the follow-up pituitary MRI showed an 8-mm lesion on the right margin of the sella turcica. With this finding, a second surgical intervention was performed and the histology reported a pituitary adenoma with mild and diffuse expression of ACTH, absence of p53 overexpression, and a Ki-67 of 2%–3%. Despite surgical rescue, the patient remained with active Cushing and an increasing tumor volume in follow-up. Thus, a third surgical intervention was performed with adjuvant stereotaxic radiotherapy at a dose of 54 Gy in 27 fractions to prevent NS but was not expected to be curative. The last histological study showed again a pituitary adenoma with ACTH expression, absence of p53 overexpression, and a Ki-67 of 2%. The patient failed to control hypercortisolism with ketoconazole (200 mg/8 h) and pasireotide (40 mg/month). Therefore, the case was presented to the endocrine tumors committee, and treatment with TBA was indicated. After TBA, the cortisol levels became undetectable and steroid replacement was initiated with hydrocortisone 300 mg iv during the first 24 h. After the initial treatment, the corticosteroid dose was gradually decreased until an oral dose of hydrocortisone at 30 mg/day was reached. Moreover, oral fludrocortisone at 0.1 mg/day was initiated. The pathological anatomy report of the adrenal glands showed diffuse corticoadrenal hyperplasia with an absence of malignancy. During postsurgical TBA follow-up, the annual pituitary MRIs showed tumor stability for 2 years.

**Figure 2:**
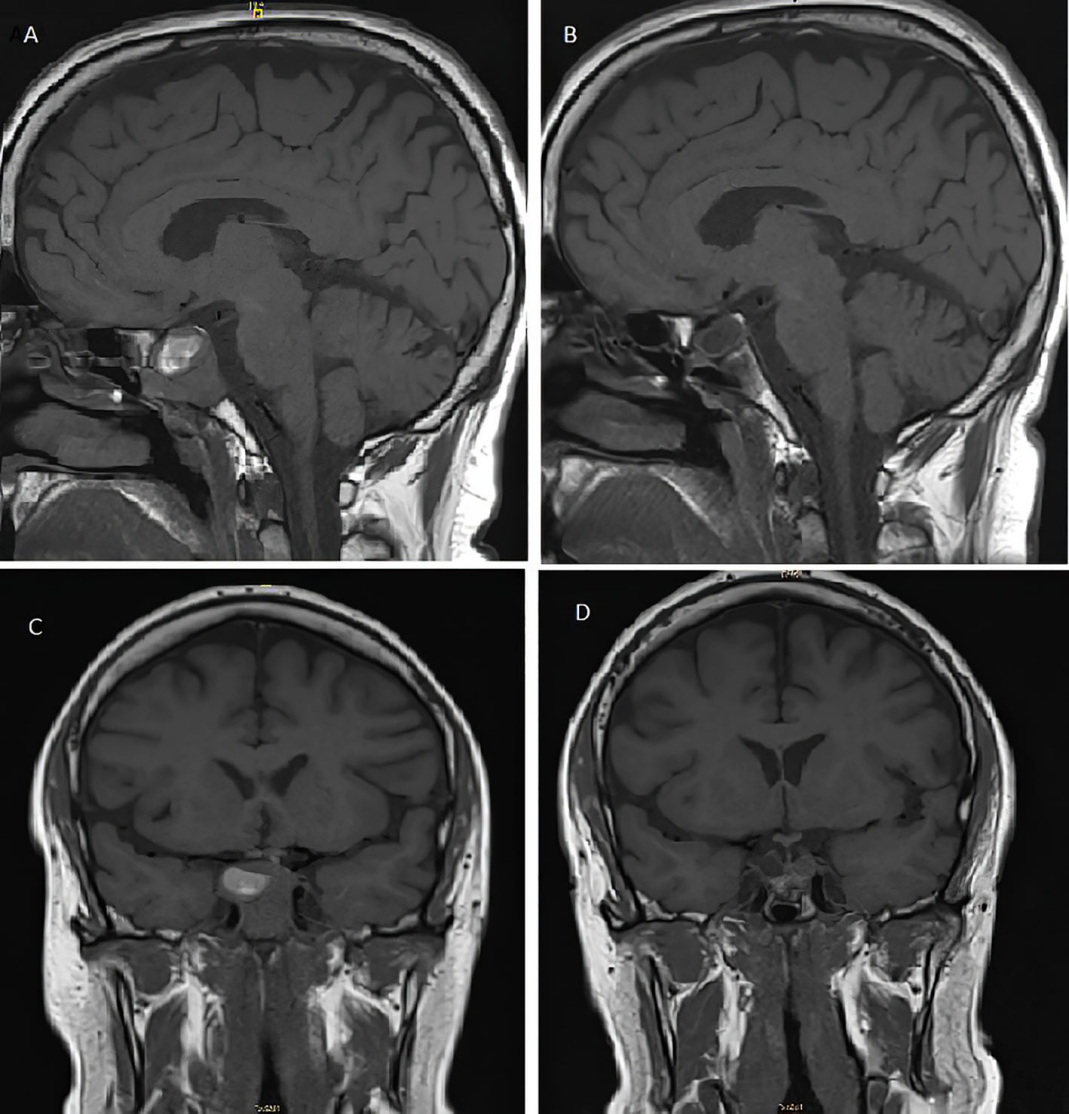
T1-weighted pituitary magnetic resonance imaging before **(A, C)** and after **(B, D)** treatment with capecitabine and temozolomide. **(A)** Pre-CAPTEM sagittal image shows an increase in the size of the seal tumor (26 mm) with a subacute bleeding component. **(B)** Post-CAPTEM sagittal image shows a decrease of 65% with a total size of 9 mm. **(C)** Pre-CAPTEM pituitary coronal image shows invasion of clivus and protrusion into the sphenoid sinus. **(D)** Post-CAPTEM coronal image shows a decrease in size of the lesion, more prominent at the right level.

**Figure 3:**
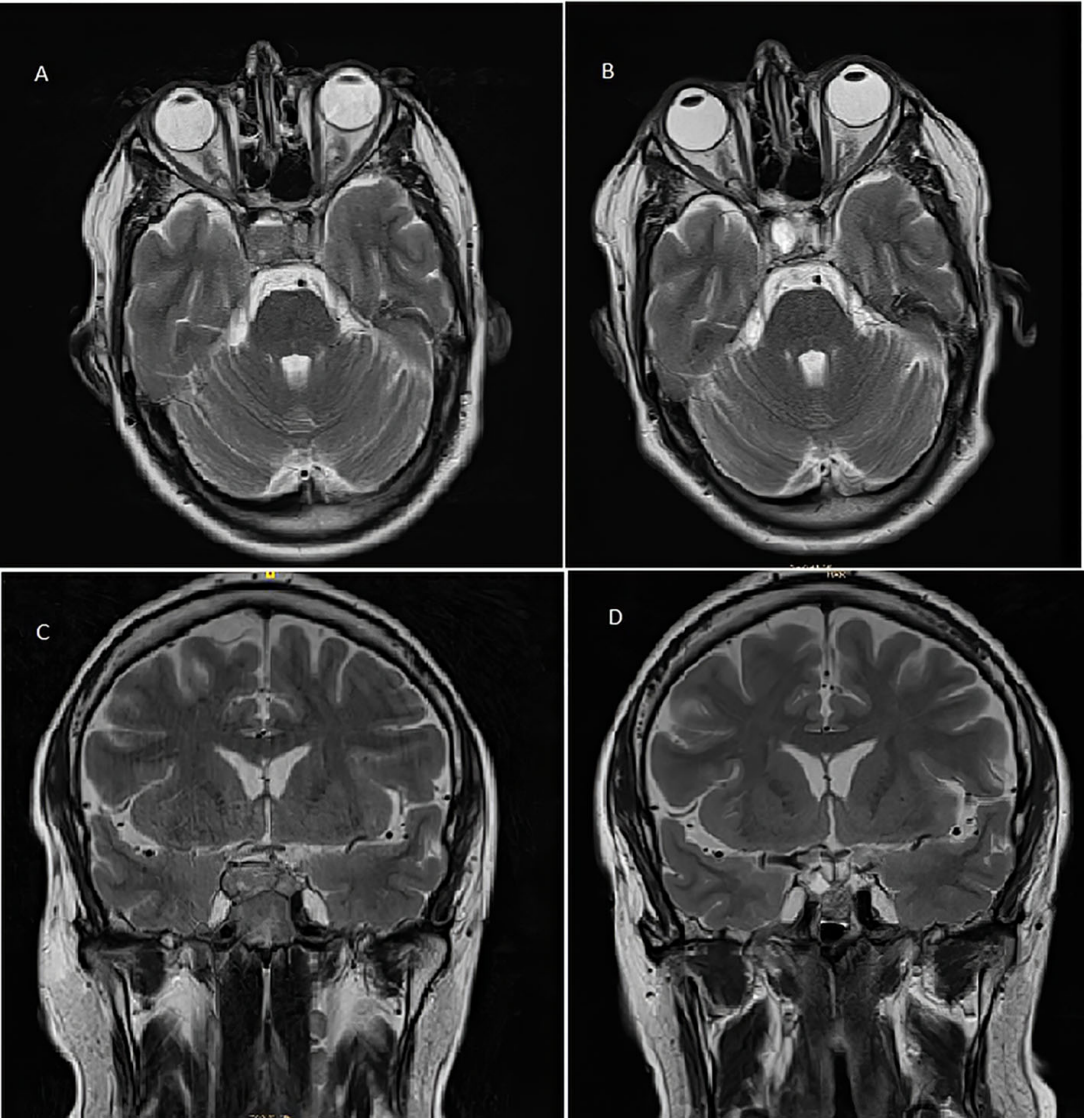
T2-weighted pituitary magnetic resonance imaging before **(A, C)** and after **(B, D)** treatment with capecitabine and temozolomide. **(A)** Pre-CAPTEM axial image. **(B)** Post-CAPTEM axial image shows a decrease in size of 65% with prominent cystic degeneration. **(C)** Pre-CAPTEM pituitary coronal image. **(D)** Post-CAPTEM coronal image shows a decrease in lesion.

Two years and eleven months after the TBA, the patient presented to the emergency room with an intermittent bilateral diplopia of 3 weeks of evolution. He denied ocular symptoms like exudate, chemosis, or ecchymosis. He also denied fever, bulbar symptoms, nausea, vomiting, headache, or head trauma. On physical examination, he presented generalized skin and mucosa hyperpigmentation, horizontal diplopia to dextroversion, without ptosis or clear restrictions in the oculomotor muscles, and no peripheral sensory or motor neurological alterations were present. The ophthalmic funduscopy and ultrasound biomicroscopy were unremarkable. The presentation of diplopia to dextroversion as the main clinical sign suggested differential diagnoses such as lens ectopias, cataracts, and corneal opiates. The ocular fundus examination without pathological findings helped to dismiss these possible diagnoses. The diplopia was not accompanied by ocular exudate, chemosis, or ecchymosis, which made it unlikely to relate the symptom to an infectious process. The neurological evaluation with an absence of peripheral sensory or motor alterations made diagnoses such as multiple sclerosis or Eaton-lambert syndrome very unlikely. Diplopia was not associated with systemic symptoms such as fever or chills, which could suggest an orbital or brain abscess or cavernous sinus thrombosis. An emergency CT scan was requested and did not show growth of the lesion or signs of compression. However, during follow-up, the patient persisted with intermittent diplopia and subsequently presented ACTH levels at 1,838 pg/ml and chromogranin A of 41.5 ng/ml (range, 0–101.9 ng/ml). A pituitary MRI (**Figures 2A, C** **)** reported an increase in the size of the pituitary tumor that invaded the clivus, a subacute bleeding component that affected the right margin of the tumor (9 × 14 mm), and an invasion of both cavernous sinuses extending to the lateral carotid line (Knosp grade III). With the three criteria of plasma ACTH level increase, imaging evidence of pituitary mass enlargement, and hyperpigmentation, the patient was diagnosed with NS. No other surgery was performed due to the low probability of success and no clear clinical benefit. Prior to the committee, a thoracic and abdominal CT scan was performed and was negative for extracranial disease. The case was presented to the endocrine tumors committee and the patient started treatment with CAP 2,500 mg daily on days 1 to 14 every 28 days for 14 days and TMZ 140 mg/m^2^ once daily on days 10 to 14 every 28 days. After two cycles of CAP-TMZ, the diplopia disappeared, hyperpigmentation improved, and ACTH levels decreased to 80% (from 1,838 to 414 pg/ml). The patient reported asthenia and diarrhea grade one during the first two cycles, which disappeared after four cycles. The patient completed four cycles of CAP-TMZ and started maintenance treatment with TMZ 140 mg/m^2^ once daily on days 10 to 14 every 28 days. After 14 months of initial CAP-TMZ treatment, the last pituitary MRI showed a 65% shrinkage of the tumor (**Figures 2** and **3B, D** **)** compared with the prior brain MRI (**Figures 3A, C** **)**. At the time of this article’s publication, after 18 months of the first dose of CAP-TMZ, the patient continues treatment with hormone replacement therapy and TMZ with excellent tolerance, maintaining a PS ECOG of 0 without new neurological focality in the last follow-up visit (**Table 1**).

**Table 1:** Treatment timeline.

Months		Case evolution
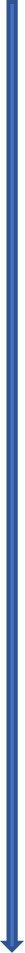	0	Diagnosis of pituitary macroadenoma
	0	First transsphenoidal endoscopic resection + biopsy
	21	Recurrence: second transsphenoidal endoscopic resection + biopsy
	29	Recurrence: third transsphenoidal endoscopic resection + biopsy + adjuvant stereotaxic radiotherapy (54 Gy in 27 fractions)
	29	Ketoconazole (200 mg/8 h)
		Pasireotide (40 mg/month)
	33	Total bilateral adrenalectomy
	68	Diagnosis of Nelson’s syndrome
	72	CAP 2,500 mg daily on days 1 to 14 every 28 days for 14 days and TMZ 140 mg/m^2^ once daily on days 10 to 14 every 28 days. (Total 4 cycles)
	76	Maintenance treatment with TMZ 140 mg/m^2^ once daily on days 10 to 14 every 28 days
	90	Follow-up: 18 months with good tolerance since first dose of CAP + TMZ treatment

CAP, capecitabine; TMZ, temozolomide.

## Discussion

The choice of treatment for a complicated case of NS is challenging due to the inability to predict a good outcome. Like in the case of our patient, Nelson’s syndrome usually presents with clinical manifestations of an enlarging pituitary tumor, including visual and cranial nerve alterations, due to tumor compressive effects or invasion into surrounding structures (6). To gain a rapid reduction in tumor size and ACTH secretion, pituitary surgery with maximal resection of the adenoma is the first-line treatment for NS, but when this approach is dismissed, there is no specific standardized systemic treatment (10). Regarding the chemotherapy options for the treatment of aggressive pituitary adenomas (APA), the use of TMZ has been described since 2006. TMZ exerts its cytotoxic activity by alkylating DNA at the O6-methylguanine DNA methyltransferase (MGMT) position of guanine resulting in irreversible DNA damage and cell death (11). Principal controversies to determine the adequate use of TMZ in patients with APA are related to the optimal duration of treatment and whether TMZ should be used in combination with other therapies. Based on the results of a European Society of Endocrinology survey in 2016, TMZ was endorsed as first-line chemotherapeutic treatment of APA (8). A previous case of a 64-year-old woman with NS treated with TMZ reported a significant improvement in symptoms, a reduction of plasma ACTH, and regression of tumor on magnetic resonance imaging scan after four cycles of treatment (12). Another review describes at least 11 separate cases of pituitary tumors treated with TMZ with sustained effects (12). In general, clinically functioning tumors and concurrent radiotherapy are associated with a better response to treatment with TMZ (8). A systematic review of 31 cases of APA reported that the objective response rate (complete response plus partial response) was 48.4%, and a stable disease occurred in 29%, and lack of response to TMZ was 22.5%. Among patients who received more than 12 months of treatment with TMZ, the majority stayed free of disease progression for relatively long periods of time (14 to 120 months). Although these data strongly argue for use of long-term TMZ in the treatment of APA, the duration of TMZ therapy remains unknown (13). A literature review that included 31 patients reported that 25 patients (80.6%) had disease control during TMZ treatment, while six patients (19.4%) had disease progression with a median follow-up after beginning TMZ of 43 months. The 2-year progression-free survival was 47.7% (95% CI, 29.5%–65.9%), while the 2-year disease control duration was 59.1% (95% CI, 39.1%–79.1%) (14).

The results of the combination of CAP-TMZ for the treatment of NS remain uncertain due to the lack of current evidence. CAP is metabolized to 5-fluorouracil and interferes with DNA synthesis and replication. Its use can lead to adverse reactions like bone marrow suppression, diarrhea, hand-foot syndrome, nausea, or fatigue (11). A synergism between CAP-TMZ has been hypothesized due to a reduction in thymidine levels (12). Previous reports that could support this theory include the case of a 48-year-old man with an aggressive corticotrope tumor who was treated with 12 cycles of CAP-TMZ, leading to tumor shrinkage and no tumor regrowth after 22 months of therapy cessation (15). A case series of four patients with aggressive ACTH producing pituitary tumors were treated with CAP-TMZ; two out of four patients demonstrated complete regression of the disease, one patient had a 75% regression, and one had an ongoing stable disease for 4.5 years at the time of the report (9). In our case, a neurological and radiological partial response was observed after 18 months of starting treatment with CAP-TMZ. These data suggest that CAP-TMZ may be a promising option for the treatment of NS with a low toxicity profile. However, some limitations must be considered. This was a single reported case; we cannot specifically determine whether the initial clinical and radiological effect of the treatment was due to the combination of CAP-TMZ or to one of the agents by themselves. The known cases of APA tend to become malignant after several years, so we cannot yet indicate if this will be the evolution of our patient. The patient received CAP-TMZ as the best possible medical treatment, according to our expertise, but from the patient’s perspective, he would prefer a drug that does not cause fatigue and allows him to go back to work. It is important to always keep in mind that the ideal treatment should be the most effective, best tolerated, and least harmful.

APA can exhibit histological features like increased proliferation, a Ki-67 index above 3%, increased mitotic cells, and p53 expression. However, the presence of these features does not predict future aggressive behavior, and the prognostic value of these markers is controversial (8). There is no correlation between the Ki-67 index and response to treatment with TMZ. Otherwise, the negative expression of the enzyme MGMT was associated with TMZ response among patients with APA (13). The MGMT repairs DNA and counteracts the effect of TMZ (8). All pathology reports of our patient showed low mitosis indicators, absence of overexpression of p53, and low KI-67 levels. MGMT expression was not available, but it might be negative due to a good response to treatment. Although the response to TMZ has been favorable for our patient, its use may limit other experimental therapies in the future. A recent study reported that peptide receptor radionuclide therapy (PRRT) can induce tumor shrinkage and clinical or biochemical improvement in 33% of patients with APA, but PRRT failure was significantly associated with previous TMZ treatment, noting that PRRT could be effective in 80% of patients not previously treated with TMZ (7).

In our case, the short latency between the tumor progression and the last session of radiotherapy, a single exposure to adjuvant stereotaxic radiotherapy with a total dose of 54 Gy, and the fact that it was a young patient make it unlikely that adjuvant radiotherapy was directly responsible for the tumor progression or a change of aggressiveness (16). Despite local tumor progression, the patient did not show new lesions at other locations of the central nervous system nor extracranial metastasis by CT scan. Thus, we treated a benign tumor with CAP-TMZ because it was rapidly growing and locally compressive. A future problem to consider is that by exposing this kind of tumor to chemotherapeutic pharmacological stress, there is a possibility of creating mechanisms of cellular resistance, mutations, and thus, malignancy. A case of a 42-year-old man with ACTH-secreting pituitary tumor evolved to a carcinoma in which the tumor progressively increased from 2.2 to 31.1 cm^3^, Ki-67 increased from 2% to 18%, and an intradural metastasis at the foramen magnum was detected. Despite these findings, the tumor presented a 90% reduction after five cycles of TMZ (200 mg/m^2^/day during the first cycle and 150 mg/m^2^/day during the following cycles) (17). Another case involved a 46-year-old woman with an APA being treated with CAP-TMZ and showing a complete biochemical and radiological response by MRI after 10 cycles. At first, each cycle was every 28 days, and then they were subsequently extended to every 42 days and every 3 months. After 8 years of treatment, the patient progressed biochemically and developed liver metastases. Due to insufficient tumor from liver biopsy, whole-exome sequencing of recurrent sellar tumor and a matched normal tissue was performed. This tumor was found to be hypermutated in the absence of microsatellite instability or mismatch repair deficiency (11). Another case of a 50-year-old man with a giant invasive corticotrope pituitary tumor treated with CAP-TMZ also presented a decrease in size and ACTH levels, but the tumor recurred after 5 months with increased avidity on PET scan, suggesting a transformation to a more aggressive phenotype (12).

All these data suggest that treatment with CAP-TMZ could be an effective option for patients with APA and NS, but treatment should be properly supervised in order to avoid secondary effects. Additionally, the use of CAP-TMZ could be explored as neoadjuvant treatment of pituitary tumors to achieve shrinkage of the tumor before surgery if complete extirpation is impossible (9). The limited or unknown long-term effect of treatment with CAP-TMZ in NS resistant to standard treatment modalities highlights the need to identify additional effective therapies.

## Patient’s Perspective

When they told me I had to receive chemotherapy, I did not take it too well, I was not amused at all, I wished there were other treatment options; however, they explained to me that it was very risky to have surgery again. I am the first person in my family that has problems with the pituitary gland, and everything that has happened to me from the beginning caught me off guard. The chemotherapy treatment has been very strong; it made me feel very tired. The first thing I noticed when starting the chemotherapy treatment was burning, pain, and erythema in the palms and soles of my hands and feet, later it made me feel very discouraged. In the aspects of my personal life, I think that the hormonal disorders and symptoms in my body increased after my pituitary gland was “fried” with radiotherapy. Sometimes, I have been discouraged because I do not feel like being intimate with my girlfriend due to my hormonal disorder. At this moment, I am following all the indications and hormonal treatments that my doctors have told me to do, in order to make these symptoms disappear. Regarding the good aspect of the treatment, I noticed that after 2 months of chemotherapy, the double vision that I was experiencing totally disappeared. I also noticed an improvement in the spots and color of my skin. In general, this is not a treatment that I would like to follow all my life, and I hope in the future there may be some other treatment options that do not make me feel tired and that allow me to return to my work and the activities that I used to do.

## Permission to Reuse and Copyright

An abstract of the case participated in the 13th SEOM MIR Clinical Case Contest and its intellectual property rights were assigned to SEOM, who authorizes the present publication. The current updated version of this case includes new content, the patient’s perspective, and posttreatment MRI images that have not been published before.

## Data Availability Statement

The original contributions presented in the study are included in the article/**Supplementary Material**. Further inquiries can be directed to the corresponding author.

## Ethics Statement

Written informed consent was obtained from the minor(s)’ legal guardian/next of kin for the publication of any potentially identifiable images or data included in this article.

## Author Contributions

OM and FF-A were major contributors in the literature review and writing the manuscript. All authors contribute to the discussion of the case. AA contributed with image description. EM-S contributed with pathological anatomy description. BB, JH, and JC contributed with their expert review of the manuscript. All authors contributed to the article and approved the submitted version.

## Conflict of Interest

The authors declare that the research was conducted in the absence of any commercial or financial relationships that could be construed as a potential conflict of interest.

## Publisher’s Note

All claims expressed in this article are solely those of the authors and do not necessarily represent those of their affiliated organizations, or those of the publisher, the editors and the reviewers. Any product that may be evaluated in this article, or claim that may be made by its manufacturer, is not guaranteed or endorsed by the publisher.
