# Supplementary material for: Aggressive Pituitary Macroadenoma Treated With Capecitabine and Temozolomide Chemotherapy Combination in a Patient With Nelson’s Syndrome: A Case Report
Source: Front Endocrinol (Lausanne). 2021 Nov 11;12:731631. doi: 10.3389/fendo.2021.731631 (PMC8632214; doi:10.3389/fendo.2021.731631)
Supplement: Supplementary file 1 [file Table_1.docx]

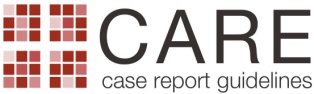
CARE Checklist of information to include when writing a case report
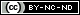


**Topic Item Checklist item description Reported on Line**

**Title 1** The diagnosis or intervention of primary focus followed by the words “case report” 1

**Key Words 2** 2 to 5 key words that identify diagnoses or interventions in this case report, including "case report" 14

Abstract

**(no references)**

**3a** Introduction: What is unique about this case and what does it add to the scientific literature? 22-24, 52

**3b** Main symptoms and/or important clinical findings 80-84

**3c** The main diagnoses, therapeutic interventions, and outcomes 88-90

**3d** Conclusion—What is the main “take-away” lesson(s) from this case? 99-102

**Introduction 4** One or two paragraphs summarizing why this case is unique (**may include references**) 138-142

**Patient Information 5a** De-identified patient specific information 174

**5b** Primary concerns and symptoms of the patient 79-84

**5c** Medical, family, and psycho-social history including relevant genetic information 56

**5d** Relevant past interventions with outcomes 59,66,69

Clinical Findings

**Timeline**

**Diagnostic Assessment**

**Therapeutic Intervention**

**Follow-up and Outcomes**

1. Describe significant physical examination (PE) and important clinical findings 82
2. Historical and current information from this episode of care organized as a timeline 55-103

**8a** Diagnostic testing (such as PE, laboratory testing, imaging, surveys). 84-91

**8b** Diagnostic challenges (such as access to testing, financial, or cultural) 84-85

**8c** Diagnosis (including other diagnoses considered) 88

**8d** Prognosis (such as staging in oncology) where applicable NA

**9a** Types of therapeutic intervention (such as pharmacologic, surgical, preventive, self-care) 59-94

**9b** Administration of therapeutic intervention (such as dosage, strength, duration) 94-96

**9c** Changes in therapeutic intervention (with rationale) 92-94

**10a** Clinician and patient-assessed outcomes (if available) 96-100

**10b** Important follow-up diagnostic and other test results 73, 86-88, 99

**10c** Intervention adherence and tolerability (How was this assessed?) 96,100,101,137

**10d** Adverse and unanticipated events NA

**Discussion 11a** A scientific discussion of the strengths AND limitations associated with this case report 137,140-142,149

**11b** Discussion of the relevant medical literature **with references** 103

**11c** The scientific rationale for any conclusions (including assessment of possible causes) 103-165

**11d** The primary “take-away” lessons of this case report (without references) in a one paragraph conclusion 166

**Patient Perspective 12** The patient should share their perspective in one to two paragraphs on the treatment(s) they received 173

**Informed Consent 13** Did the patient give informed consent? Please provide if requested . . . . . . . . . . . . . . . . . . . . . . . . . . . . . . . . . . . . . . **Yes No**
